# Diagnosis, treatment and prognosis of small bowel volvulus in adults: A monocentric summary of a rare small intestinal obstruction

**DOI:** 10.1371/journal.pone.0175866

**Published:** 2017-04-20

**Authors:** Xiaohang Li, Jialin Zhang, Baifeng Li, Dehui Yi, Chengshuo Zhang, Ning Sun, Wu Lv, Ao Jiao

**Affiliations:** Department of General Surgery, First Affiliated Hospital, China Medical University, Shenyang, Liaoning Province, China; University Hospital Llandough, UNITED KINGDOM

## Abstract

**Objectives:**

Small bowel volvulus is a rare disease, which is also challenging to diagnose. The aims of this study were to characterize the clinical and radiological features associated with small bowel volvulus and treatment and to identify risk factors for associated small bowel necrosis.

**Methods:**

Patients with small bowel volvulus who underwent operations from January 2001 to December 2015 at the First Affiliated Hospital of China Medical University (Shenyang, China) were reviewed. Clinical, surgical and postsurgical data were registered and analyzed.

**Results:**

Thirty-one patients were included for analysis. Fifteen patients were female (48.4%), with an average age of 47.7 years (18–79 years). The clinical signs and symptoms were unspecific and resembled intestinal obstruction. Clinical examination revealed abdominal distension and/or diffuse tenderness with or without signs of peritonitis. The use of CT scans, X-rays or ultrasound did not differ significantly between patients. In 9 of 20 patients that received abdominal CT scans, “whirlpool sign” on the CT scan was present. Secondary small bowel volvulus was present in 58.1% of patients, and causes included bands (3), adhesion (7), congenital anomalies (7) and stromal tumor (1). Out of the 31 patients, 15 with gangrenous small bowel had to undergo intestinal resection. Intestinal gangrene was present with higher neutrophils count (*p*<0.0001) and the presence of bloody ascites (*p* = 0.004). Three patients died of septic shock (9.68%), and the recurrence rate was 3.23%.

**Conclusions:**

To complete an early and accurate diagnosis, a CT scan plus physical exam seems to be the best plan. After diagnosis, an urgent laparotomy must be performed to avoid intestinal necrosis and perforation. After surgery, more than 90% of the patients can expect to have a favorable prognosis.

## Introduction

Small bowel volvulus (SBV) in adults is a very rare disease [1], and presentation as a closed-loop obstruction in a patient can lead to a poor outcome. Therefore, early diagnosis and treatment of SBV is important. SBV is characterized by torsion of a segment of small bowel and its mesentery. SBV can be classified as primary or secondary SBV according to the cause. Primary SBV is defined as torsion of a segment of small bowel at the mesentery basis without any evident underlying cause [2]; secondary SBV occurs in the presence of an acquired condition, such as congenital malrotation, anatomic abnormality, bands, postoperative adhesions, tumors, pregnancy, and diverticular disease [3]. No specific symptom, clinical sign, or abnormal laboratory finding covers both primary and secondary types [4]. As the abdominal imaging cannot always provide enough imaging information for the diagnosis, a delay in diagnosis may occur, which can be life-threatening.

Early diagnosis and prompt operation are essential to prevent gangrene in the small intestine, which is associated with high morbidity and mortality. Although some papers have been published on SBV, most of these were case reports [5–7]. In fact, no previous reports in the literature have described risk factors associated with necrosis in SBV cases.

In this study, we describe our experience associated with SBV and report details of 31 patients, including symptoms, etiological factors, abdominal imaging, treatment, and clinical outcomes. We also analyze the risk factors associated with small bowel necrosis in SBV, so that surgeons can diagnose and treat this disease timely and appropriately. All physicians should be aware of this rare but dangerous disease to offer patients the best management available.

## Materials and methods

All data were collected under protocols approved by the institutional review board of China Medical University. We performed a retrospective study of 31 patients with small bowel volvulus who underwent operations from January 2001 to December 2015 at our institution in Shenyang, China. We analyzed clinical features—such as age, gender, previous surgery, clinical signs, imaging studies, preoperative diagnosis, intraoperative findings, mortality, and recurrence—to provide surgeons with the best evidence for optimal treatment. We divided the patients with SBV into two groups—a group with necrosis and one without—and different variables were compared between the two groups to identify risk factors associated with small bowel necrosis in SBV. We conducted this study between February and April in 2016.

The mean ± standard deviation (SD) and range of continuous variables were calculated and are presented in Tables 1–4. Categorical variables were defined by whole number and percentage. The statistical significance of the differences between groups was determined by *t*-test or χ^2^ test. A *p* value <0.05 was considered statistically significant.

**Table 1:** Categorization of the small bowel volvulus.

Reason	No.	Percentage (%)
Primary	13	41.9
Secondary		
Adhesion	10	32.3
Stromal tumor	1	3.2
Bowel malrotation	4	12.9
Mesenteric hiatal hernia	3	9.7

**Table 2:** Clinical manifestations and blood routine tests of the 31 patients with small bowel volvulus.

	No.	Percentage (%)
Disease course less than 24 hours	16	51.6%
Sudden abdominal pain	30	96.8%
Nausea/vomiting	20	64.5%
Abdominal distention	14	45.2%
No passing of gas or feces	20	64.5%
Peritonitis	19	61.3%
Bowel sounds absence	17	54.8
An elevated ratio of neutrophils	22	71.0%

**Table 3:** Statistical analysis of clinical characteristics between small bowel volvulus with and without the necrotic intestine.

Variables	SBV with the necrotic intestine (n = 17, 54.8%)	SBV without the necrotic intestine (n = 14, 45.2%)	P (Fisher’s exact test probabilities)
Pattern			
Primary	7	6	1
Secondary	10	8	
Direction			
Clockwise	9	8	1
Counter clockwise	8	6	
Disease course			
Less than 24 h	12	4	**0.032**
More than 24 h	5	10	
Sign			
Peritonitis	11	8	0.724
Non-peritonitis	6	6	
Bloody ascites			
Ascites	13	1	**<0.0001**
Without ascites	4	13	
Bowel sounds			
Weak bowel sound	11	6	0.289
High-pitched bowel sound	6	8	
Ratio of neutrophils			
Raised ratio of neutrophils	16	6	**0.004**
Normal ratio of neutrophils	1	8	

**Table 4:** Surgical procedures performed in the 31 patients.

	No.	Percentage (%)
Laparotomy alone	2	6.45%
Bowel resection	13	48.4%
Bowel resection + mesenteric hiatal hernia repair	2	6.45%
Resection of small bowel stromal tumor	1	3.23
Simple devolvulation	5	16.1%
Devolvulation + adhesiolysis	5	16.1%
Devolvulation + decompression	2	6.45
Devolvulation + mesenteric hiatal hernia repair	1	3.23%

## Results

Thirty-one patients were included in our study comprising 16 males (51.6%) and 15 females (48.4%) with a mean age of 47.7 ± 15.5 years (range from 18–79 years). Most of the patients were diagnosed with acute intestinal obstruction before surgery, and we made a correct preoperative diagnosis in only 9 of the 31 patients (29%). Primary and secondary SBV were identified in 41.9% (13) and 58.1% (18) patients, respectively. Causes of secondary small bowel volvulus included bands (3 patients), adhesion (7 patients), anatomic abnormality (3 patients), congenital anomalies (4 patients), and stromal tumor (1 patient) (Table 1).

There was a wide range in the duration of symptoms (4 hours to 1 week) before the patients presented at the emergency department. The most common symptom was abdominal pain followed by nausea/vomiting and passing no gas or feces. There were 19 patients with peritonitis signs, 16 patients with symptoms lasting less than 24 hours, and 17 patients without bowel sounds. Laboratory tests indicated neutrophilia in 22 patients (71%) with more than 75% of neutrophils (Table 2).

**Fig 1:**
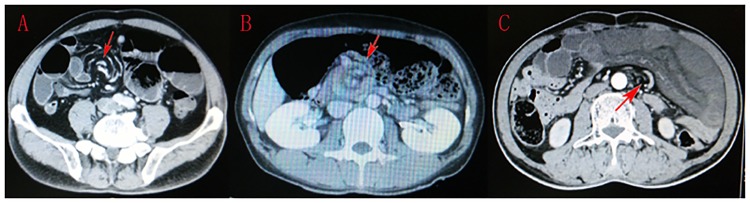
Contrast-enhanced abdominal CT demonstrated the dilation of small bowels and whirl-like pattern of distended small bowels encircling the mesenteric artery or its branches (red arrows).

**Fig 2:**
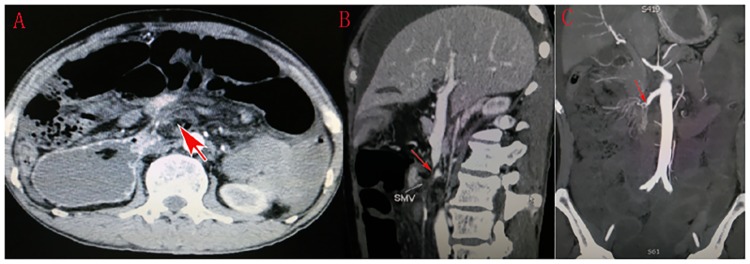
Three-dimensional abdominal CT showed the whirl sign of superior mesenteric vessels (A, red arrow). The longitudinal tapering of superior mesenteric vein (B) and artery (C) were found in the coronal scan.

Abdominal X-rays, abdominal sonography and CT scans were the three main imaging tests used in this study. Plain abdominal X-rays were performed in 21 patients; it was the most frequently used imaging technique but the least sensitive diagnosis test. Abdominal sonography only revealed the distended intestine and reverse peristalsis, and could not identify the causes leading to intestinal obstruction. Abdominal CT scans were performed in 20 patients, and the characteristic whirlpool sign (a tightly twisted mesentery around the point of torsion) presented in 9 cases (Figs 1 and 2); hence abdominal CT scans diagnosed SBV with 45% accuracy.

Unfortunately, our study could not acquire the exact evolution time from admission to operation. Intraoperatively, there were 17 examples of a clockwise rotation of small bowel volvulus, and the rest were counter clockwise. Small intestinal necrosis was observed in 17 patients (54.8%), and 13 of those patients presented with massive bloody ascites. Moderately bloody ascites was observed in only 1 of the 31 patients, and small intestinal necrosis was not observed in that patient.

There was no significant difference in the incidences of gut necrosis between patients grouped by small intestinal volvulus pattern, rotation direction, peritonitis presence, and bowel sound status (all *p*>0.05), but there was a significant difference between patients grouped by disease course (*p* = 0.032), ratio of neutrophils (*p*<0.0001) and presence of bloody ascites (*p* = 0.004) (Table 3). However, longstanding symptoms are more likely to be caused by intermittent volvulus, which is less likely to cause precipitating ischemia, whereas a sudden complete mesenteric torsion will do so. The conclusion that symptoms lasting less than 24 hours will result in gangrenous bowel is a misinterpretation of these findings. Therefore, we considered that higher ratio of neutrophils and presence of bloody ascites were associated with intestinal necrosis.

Surgical procedures are described in Table 4. In our study, except for simple devolvulation (5 cases), simultaneous adherensiolysis (5 cases), decompression (2 cases) and mesenteric hiatal hernia repair (1 case) were performed according to each patient’s pathogeny. Three patients presented an internal hernia with secondary volvulus; the patient without necrosis was managed with devolvulation and the others with necrosis required resection. Four patients presented with intestinal malrotation, one managed with intestinal resection and three with devolvulation. One patient presented with a stromal tumor of the jejunum complicated with SBV, and intestinal resection was performed. Two patients presented with widespread necrosis of the small bowel and only a laparotomy was performed in those cases.

After surgery, only three patients presented with complications: the two with necrosis in almost all their small bowel and one with septic shock on the eleventh postoperative day. The overall mortality rate was 9.68%. SBV recurred in one patient (3.23%) 6 months later.

## Discussion

The incidence of SBV is high (24–60 per 100,000 people) in Middle Eastern, Asian and Central African countries, but low (1.7–5.7 per 100,000 people) in Western countries [8]. While the exact reason for these regional differences remains unknown, it perhaps relates to dietary factors. Fiber consumption after prolonged fasting (e.g. Muslims during Ramadan) results in sudden overloading of the empty bowel, which can induce bowel peristalsis thus leading to SBV [9]. However, the literature in some Asian countries such as Japan and Taiwan showed that SBV was rare in those countries [10,11]. It is usually difficult to diagnose SBV preoperatively; nevertheless, compared to primary SBV, it may be easier to diagnose secondary SBV as some pathologic factors such as adhesions, small bowel tumors and Meckel's diverticulum cause us to suspect secondary SBV.

SBV is a rare cause of small intestinal obstruction, and it is often not diagnosed before surgery. Symptoms of SBV and other causes of intestinal obstruction are very similar. Indeed, abdominal X-rays and ultrasound scans could not clearly show any specific findings in most cases; for this reason, a CT scan must be performed in suspected cases of SBV. In our article the diagnostic rate by abdominal contrast CT scan was 45%, which corresponds well with findings reported by other authors such as Huang whose rate was 52.6% [11].

There were 17 patients with small bowel necrosis, and the length of necrotized bowel ranged from 8 cm to 200 cm. We compared some clinic signs and risk factors between the two groups: one with small bowel necrosis and one without. We found that with primary or secondary SBV, direction of rotation, presence of peritonitis, and bowel sound status were not associated with bowel necrosis. The presence of some pathologic factors—such as congenital malrotation, bands, tumor, and postoperative adhesion—was suggested as the main etiologic reason behind secondary SBV. However, there was no specific associated cause of primary SBV. Therefore, the preoperative diagnosis of primary SBV is more difficult than secondary SBV.

In theory, a delayed diagnosis occurs more often in primary SBV, and consequently bowel necrosis is more likely. Our article did not support this result, however, the possible reason being that the diagnostically useful abdominal contrast CT scan was performed in a timely fashion when the SBV was suspected. We also found that the clockwise or counter-clockwise rotation of small bowel is not associated with intestinal necrosis.

Our primary data did not provide enough information to elucidate whether the extension of rotation is related to necrosis. What surprised us is that bowel sound status could not predict small bowel activity, as normal bowel sounds may occur in patients with small bowel necrosis too. One theory is that compensatory peristalsis of non-necrotic small bowel was present in the initial stage. Our article found that 11 (64.7%) of the 17 patients with necrotic bowel showed signs of peritoneal irritation, which closely corresponded to the reported rate of 60% in the study by Roggo and Ottinger [4]. Thus approximately 40% of patients with small bowel necrosis may not present with peritonitis.

In the present study all analyzed factors—including the course of the disease, the ratio of neutrophils and the presence of bloody ascites—showed a statistical correlation with necrotic small bowel. The duration from the first signs or symptoms to emergency admission ranged from a few hours to 7 days, and we found that small bowel necrosis even occurred within 24 hours of symptoms developing although these symptoms were often acute and severe, with patients being admitted to hospital quickly.

Patients with symptoms lasting more than 24 hours before hospital admission developed disease insidiously, and most of these did not present with small bowel gangrene. We found that the 22 patients with elevated neutrophils ratios comprised only 6 of the 14 patients (43%) with viable bowel but 16 of the 17 patients (94%) with necrotic bowel. Therefore, it appears that an elevated neutrophils ratio closely correlates to small bowel necrosis. Moreover, venous reflux disturbance may occur in SBV patients, and ascites may gradually emerge. Indeed, yellow and clear ascites are common in the early stage of the disease. When the small bowel becomes necrotic, the ascites seems bloody and the volume is often large. Therefore, the presence of bloody ascites may indicate intestinal necrosis, and thus a laparotomy should be performed if this is present in the paracentesis.

Once a diagnosis of SBV is suspected, an emergency operation must soon be performed to prevent new development of small bowel necrosis. The exact surgical procedure chosen depends on the viability of the small bowel, the abdominal anatomical condition and whether there are other concomitant diseases. Simple devolvulation is frequently performed when the small bowel is viable and there are no other concomitant diseases, and small bowel resection is performed when it becomes gangrenous.

SBV may develop as a consequence of postoperative adhesions. Bowel adhesions to the parietal peritoneum may stretch the intestine and lead to its rotation. In other cases, adhesions between the intestines cause the intestines to be tied and the resulting clamped intestine presents with rotation. Therefore, laparotomy and adhesiolysis need to be performed in these patients along with intestine resection according to the small bowel’s viability. Moreover, repair is necessary if an internal hernia is found intraoperatively in order to prevent the recurrence of SBV. For patients with severe intestinal obstruction caused by SBV, decompression should be performed to alleviate intestinal tension and edema.

Among all patients included in our research only one patient received laparoscopic surgery where devolvulation and decompression were performed. Indeed, few studies in the literature report SBV treatment performed laparoscopically. Kim *et al*. reported intestinal resection done laparoscopically [12]. There are many advantages associated with laparoscopy. It is less invasive, allows faster recovery, and diagnostic laparoscopy is very useful when the diagnosis is uncertain. Therefore, we should perform laparoscopic surgery in more patients with suspected SBV.

Fortunately, no perforation of strangulated small bowel occurred in our series of cases. However, three patients died after surgery due to necrosis in the small bowel along with severe abdominal sepsis. The overall mortality was 9.68%, which was similar to previous reports (9.3%) [13].

In our research, SBV only recurred in one patient (6 months after surgery). The patient presented with primary SBV, and simple devolvulation without fixation was performed. Thus our recurrence rate was 3.23%, which was lower than the rate recorded by Ruiz-Tovar *et al*. (3.87%) [13].

There is a continuing controversy regarding the management of primary volvulus. Some authors considered that simple devolvulation of the involved bowel is the most appropriate operation [14], however others recommend additional intestinal fixation or even suggest that resection is necessary to avoid a recurrence [9,15].

We think that management of volvulus without necrosis depends on the actual condition of the individual patient, including the length of the small bowel and mesentery, and anatomy of the small bowel. If there are no anatomical abnormalities, simple devolvulation without fixation is not associated with high recurrence. Conversely, fixation or resection of the intestine could be performed depending on the surgeon’s experience, when the small bowel or mesentery is long or presents with other anatomical abnormality.

## Conclusion

SBV is a rare, acute abdominal condition, and its clinical presentation is similar to acute intestinal obstructions caused by other conditions. A delay in diagnosis can lead to disastrous results, so we should attach more importance to this disease. Among conventional abdominal imaging, the characteristic whirlpool sign in abdominal CT scan may provide the best diagnostic indicator.

Our study shows that an elevated ratio of neutrophils and the presence of bloody ascites often indicate small bowel necrosis, therefore we should pay more attention to these two aspects when treating patients with suspected SBV. Early diagnosis and appropriate surgical procedures are essential for preventing necrosis and obtaining a better prognosis.

Suitable surgical procedures include devolvulation with/without fixation, and resection of the gangrenous segment and settlement of the causes. The chosen surgical procedure depends on the surgeon’s experience and the patient’s condition. Laparoscopy also could be used to successfully treat such patients.
